# Dissecting mammalian reproduction with spatial transcriptomics

**DOI:** 10.1093/humupd/dmad017

**Published:** 2023-06-23

**Authors:** Xin Zhang, Qiqi Cao, Shreya Rajachandran, Edward J Grow, Melanie Evans, Haiqi Chen

**Affiliations:** Cecil H. and Ida Green Center for Reproductive Biology Sciences, University of Texas Southwestern Medical Center, Dallas, TX, USA; Department of Obstetrics and Gynecology, University of Texas Southwestern Medical Center, Dallas, TX, USA; Cecil H. and Ida Green Center for Reproductive Biology Sciences, University of Texas Southwestern Medical Center, Dallas, TX, USA; Department of Obstetrics and Gynecology, University of Texas Southwestern Medical Center, Dallas, TX, USA; Cecil H. and Ida Green Center for Reproductive Biology Sciences, University of Texas Southwestern Medical Center, Dallas, TX, USA; Department of Obstetrics and Gynecology, University of Texas Southwestern Medical Center, Dallas, TX, USA; Cecil H. and Ida Green Center for Reproductive Biology Sciences, University of Texas Southwestern Medical Center, Dallas, TX, USA; Department of Obstetrics and Gynecology, University of Texas Southwestern Medical Center, Dallas, TX, USA; Department of Obstetrics and Gynecology, University of Texas Southwestern Medical Center, Dallas, TX, USA; Cecil H. and Ida Green Center for Reproductive Biology Sciences, University of Texas Southwestern Medical Center, Dallas, TX, USA; Department of Obstetrics and Gynecology, University of Texas Southwestern Medical Center, Dallas, TX, USA

**Keywords:** spatial transcriptomics, reproduction, gametogenesis, pregnancy, embryogenesis, cancer

## Abstract

**BACKGROUND:**

Mammalian reproduction requires the fusion of two specialized cells: an oocyte and a sperm. In addition to producing gametes, the reproductive system also provides the environment for the appropriate development of the embryo. Deciphering the reproductive system requires understanding the functions of each cell type and cell–cell interactions. Recent single-cell omics technologies have provided insights into the gene regulatory network in discrete cellular populations of both the male and female reproductive systems. However, these approaches cannot examine how the cellular states of the gametes or embryos are regulated through their interactions with neighboring somatic cells in the native tissue environment owing to tissue disassociations. Emerging spatial omics technologies address this challenge by preserving the spatial context of the cells to be profiled. These technologies hold the potential to revolutionize our understanding of mammalian reproduction.

**OBJECTIVE AND RATIONALE:**

We aim to review the state-of-the-art spatial transcriptomics (ST) technologies with a focus on highlighting the novel biological insights that they have helped to reveal about the mammalian reproductive systems in the context of gametogenesis, embryogenesis, and reproductive pathologies. We also aim to discuss the current challenges of applying ST technologies in reproductive research and provide a sneak peek at what the field of spatial omics can offer for the reproduction community in the years to come.

**SEARCH METHODS:**

The PubMed database was used in the search for peer-reviewed research articles and reviews using combinations of the following terms: ‘spatial omics’, ‘fertility’, ‘reproduction’, ‘gametogenesis’, ‘embryogenesis’, ‘reproductive cancer’, ‘spatial transcriptomics’, ‘spermatogenesis’, ‘ovary’, ‘uterus’, ‘cervix’, ‘testis’, and other keywords related to the subject area. All relevant publications until April 2023 were critically evaluated and discussed.

**OUTCOMES:**

First, an overview of the ST technologies that have been applied to studying the reproductive systems was provided. The basic design principles and the advantages and limitations of these technologies were discussed and tabulated to serve as a guide for researchers to choose the best-suited technologies for their own research. Second, novel biological insights into mammalian reproduction, especially human reproduction revealed by ST analyses, were comprehensively reviewed. Three major themes were discussed. The first theme focuses on genes with non-random spatial expression patterns with specialized functions in multiple reproductive systems; The second theme centers around functionally interacting cell types which are often found to be spatially clustered in the reproductive tissues; and the thrid theme discusses pathological states in reproductive systems which are often associated with unique cellular microenvironments. Finally, current experimental and computational challenges of applying ST technologies to studying mammalian reproduction were highlighted, and potential solutions to tackle these challenges were provided. Future directions in the development of spatial omics technologies and how they will benefit the field of human reproduction were discussed, including the capture of cellular and tissue dynamics, multi-modal molecular profiling, and spatial characterization of gene perturbations.

**WIDER IMPLICATIONS:**

Like single-cell technologies, spatial omics technologies hold tremendous potential for providing significant and novel insights into mammalian reproduction. Our review summarizes these novel biological insights that ST technologies have provided while shedding light on what is yet to come. Our review provides reproductive biologists and clinicians with a much-needed update on the state of art of ST technologies. It may also facilitate the adoption of cutting-edge spatial technologies in both basic and clinical reproductive research.

## Graphical abstract

**Figure:**
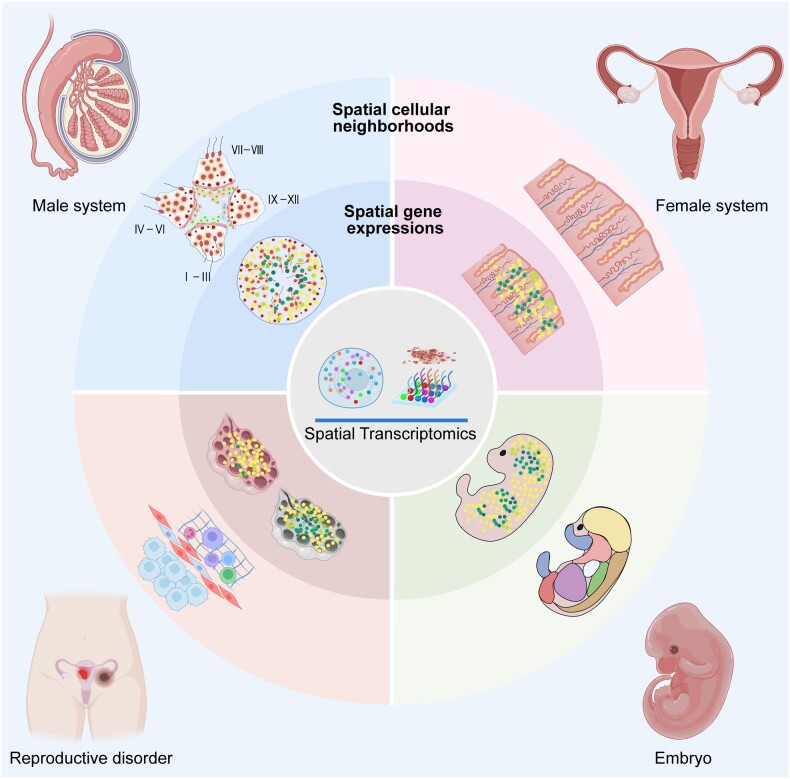
Applications of spatial transcriptomics technologies in mammalian reproductive systems in the context of gametogenesis, embryogenesis, and reproductive pathologies.

## Introduction

Reproduction ensures the transmission of genetic and epigenetic information to the next generation and the continuity of species. The maintenance of the reproductive systems, the generation of gametes, and embryonic development are some of the central focuses of reproductive biology. A deep understanding of mammalian reproduction could facilitate the diagnosis and treatment of infertility, cancer, and other reproductive pathologies, as well as the development of contraceptives.

Mammalian reproduction is regulated by numerous biological pathways and involves many cell types. For example, the development of gametes is a highly regulated process, which includes, but is not limited to, chromatin remodeling, epigenetic reprogramming, cell cycle regulation, meiosis, and cell migration (Marston and Amon, 2004; Richardson and Lehmann, 2010; Yosefzon *et al.*, 2017; Cabot and Cabot, 2018; Larose *et al.*, 2019; Fang *et al.*, 2022). It also requires cellular and molecular interactions between developing gametes and surrounding somatic cell types (Wassarman, 2002; Mruk and Cheng, 2004; Gershon *et al.*, 2008; Hofmann and McBeath, 2022). Deciphering such complexity requires technologies capable of characterizing molecular and cellular processes at scale. While recent single-cell technologies offer a high throughput solution (Vitak *et al.*, 2017; Wang *et al.*, 2018b, 2019, 2021; Green *et al.*, 2018; Guo *et al.*, 2018; Argelaguet *et al.*, 2019; Ferrero *et al.*, 2019; McGinnis *et al.*, 2019; Zhao *et al.*, 2020; Cheung *et al.*, 2021; Li *et al.*, 2018, 2021; Mittnenzweig *et al.*, 2021; Yan *et al.*, 2021; Zhang *et al.*, 2021; Garcia-Alonso *et al.*, 2022), they require tissue dissociation, which results in the loss of spatial context and significant cellular information such as cell–cell and cell–extracellular matrix interactions.

Spatial transcriptomics (ST) technologies have emerged as tools that can not only provide the information on the abundance of mRNA molecules in the cells but also capture their spatial locations within the tissue (Marx, 2021; Rao *et al.*, 2021; Tian *et al.*, 2023). These technologies range from laser capture microdissection (LCM), *in situ* hybridization (ISH) and *in situ* sequencing (ISS) to solid phase-based capturing technologies (Marx, 2021; Rao *et al.*, 2021; Tian *et al.*, 2023). Together, they play a crucial role in exploring the spatial distribution of RNA, the spatial location of cell populations, and cell–cell interactions. In this review, we introduce major ST technologies that have been applied to mammalian reproductive systems, discuss in detail the biological insights that have been revealed by studies using ST, and offer an outlook for the future of ST technologies and how they can further benefit the field of reproductive biology in the near future.

## Overview of ST technologies

ST technologies can be primarily categorized into two classes based on their design principles. The first class relies on the imaging of pre-determined mRNA targets. These targeted approaches include ISH-based methods and ISS-based methods. The second class of ST technologies includes unbiased approaches that build on spatial isolation/capture of RNA molecules followed by next-generation sequencing (NGS). For readers who are interested in the current landscape of ST technologies, we have compiled a list of representative ST technologies in Table 1. For readers who are interested in learning about computational approaches to analyze ST data, we recommend these excellent reviews (Dries *et al.*, 2021; Longo *et al.*, 2021; Zeng *et al.*, 2022). In the next two sections, we focus on ST technologies that have been applied to the reproductive systems.

**Table 1.** A list of representative spatial transcriptomics technologies.

TARGETED APPROACHES							
***In situ* hybridization (ISH)**							

**Year**	**Methods**	**Authors**	**Features**	**Number of targets**	**Spatial resolution**	**Limitations**	**Estimated cost** *

1998	smFISH (Femino *et al.*, 1998)	Andrea M. Femino	Multiple oligonucleotide probes hybridize with the same transcript.	4–5 targets	Subcellular resolution	The spectral overlap limits the simultaneous detection of multiple transcripts.	∼$120/sample; Microscope needed.
2014	seqFISH (Lubeck *et al.*, 2014)	Eric Lubeck *et al.*	The mRNA molecules are barcoded by sequential rounds of hybridization, imaging, and probe stripping.	Dozens of targets within individual cell	Subcellular resolution	Complex experimental setup: data analysis is challenging.	Custom microfluidics and flow cell: ∼$5000; consumable: ∼$500/sample; microscope needed.
2015	MERFISH (Chen *et al.*, 2015)	Kok Hao Chen *et al.*	Using the binary words of modified Hamming code to encode the RNA molecules.	∼100–1000 genes within individual cell	Subcellular resolution	Costly instrument	Commercial Vizgen MERSCOPE Instrument: ∼$300 000; consumable: ∼$600/sample.
2019	MERFISH (Xia *et al.*, 2019)	Chenglong Xia *et al.*	Enhanced throughput of the original MERFISH.	∼10 000 genes within individual cell	Subcellular resolution	Complex experimental setup; data analysis is challenging.	Custom microfluidics and flow cell: ∼$5000; consumable: ∼$500/sample; microscope needed.
2019	seqFISH+ (Eng *et al.*, 2019)	Chee-Huat Linus Eng *et al.*	Using 60 ‘pseudocolor’ channels to dilute mRNA molecules.	∼10 000 within individual cell	Sub-diffraction limit resolution	Complex experimental setup; data analysis is challenging.	Custom microfluidics and flow cell: ∼$5000; consumable: ∼$600/sample. microscope needed.
2019	GeoMix (Nanostring, 2019)	Nanostring	Based on probes linked to indexing oligo barcodes via a photocleavable linker.	A few hundred genes	Single-cell resolution	Costly instrument	Commercial GeoMix instrument: ∼$290 000; consumable: ∼$500/sample. Additional NGS required.

***In situ* sequencing (ISS)**							
**Year**	**Methods**	**Authors**	**Features**	**Number of targets**	**Spatial resolution**	**Limitations**	
2013	ISS (Ke *et al.*, 2013)	Rongqin Ke *et al.*	Based on padlock probes, RCA, and sequencing-by-ligation chemistry.	256 targets within a single cell	Subcellular resolution	Laborious and low efficiency	Commercial microfluidics and flow cell: ∼$5000; consumable: ∼$500/sample. Microscope needed.
2014	FISSEQ (Lee *et al.*, 2014)	Je Hyuk Lee *et al.*	RNA is reverse transcribed with tagged random hexamers; RCA; SOLiD sequencing	Entire transcriptome	Subcellular resolution	Low sequencing depth; time-consuming protocol; SOLiD sequencing reagents have been discontinued	Commercial microfluidics and flow cell: ∼$5000; consumable: ∼$500/sample. Microscope needed.
2018	BaristaSeq (Chen *et al.*, 2018)	Xiaoyin Chen *et al.*	Based on padlock probes and RCA; using *in situ* barcode sequencing compatible with Illumina sequencing chemistry	Entire transcriptome	Subcellular resolution	Low sequencing depth	Custom microfluidics and flow cell: ∼$5000; consumable: ∼$600/sample. Microscope needed.
2018	STARmap (Wang *et al.*, 2018a)	Xiao Wang *et al.*	Based on SNAIL probes, RCA, and fluorescent *in situ* sequencing	Detecting ∼1000 transcripts in a cell	Subcellular resolution	Complex experimental setup; data analysis is challenging.	Custom microfluidics and flow cell: ∼$5000; consumable: ∼$600/sample. Microscope needed.
2021	ExSeq (Alon *et al.*, 2021)	Shahar Alon *et al.*	Based on expansion microscopy	3039 genes	Nanoscale resolution	Complex experimental setup; data analysis is challenging.	Custom microfluidics and flow cell: ∼$5000; consumable: ∼$600/sample. Microscope needed.
2023	Xenium (10× Genomics, 2023)	10× Genomics	Padlock probe-based RCA and fluorescent *in situ* sequencing.	300 genes	Subcellular resolution	Costly instrument	Commercial Xenium instrument: ∼$350 000; consumable: ∼$1000/sample.

**UNBIASED APPROACHES**							

**Year**	**Methods**	**First author**	**Features**	**Number of targets**	**Spatial resolution**	**Limitations**	**Estimated cost**

1996	LCM (Emmert-Buck *et al.*, 1996)	Michael R. Emmert-Buck *et al.*	Using laser to accurately obtain target cell subgroups or single cells	Entire transcriptome	Single-cell resolution	Low throughput; time-consuming protocol	Commercial LCM instrument: ∼$150 000; consumable: ∼$100/sample; additional NGS required.
2016	Spatial Transcriptomics (Ståhl *et al.*, 2016)	Patrik L. Ståhl *et al.*	Capturing RNA using spatially indexed poly(dT) oligo arrays.	Entire transcriptome	100 µm	Low sensitivity for low abundant transcripts; low spatial resolution	∼$650/sample; additional NGS required.
2017	GEO-seq (Chen *et al.*, 2017)	Jun Chen *et al.*	Combining LCM with single-cell RNA-seq	Entire transcriptome	Single-cell resolution	Low throughput; time-consuming protocol	Commercial LCM instrument: ∼$150 000; consumable: ∼$1000/sample; additional NGS required.
2019	Visium (10× Genomics, 2019)	10× Genomics	Based on the spatial transcriptomics technology	Entire transcriptome	∼55 μm	Low spatial resolution	∼$3000/sample; additional NGS required.
2019	Slide-seq (Rodriques *et al.*, 2019)	Samuel G. Rodriques *et al.*	Capturing mRNA using spatially indexed 10-μm-diameter beads	Entire transcriptome	10 μm	Low sensitivity for low abundant transcripts	∼$1000/sample; additional NGS required.
2020	DBiT-seq (Liu *et al.*, 2020)	Yang Liu *et al.*	Using microfluidic chip and DNA barcodes to spatially index molecules in the tissue	Entire transcriptome	10 μm	Requiring a custom microfluidic device	Custom microfluidics: ∼$7000; consumable: ∼$1400/sample; additional NGS required.
2021	Slide-seqV2 (Stickels *et al.*, 2021)	Robert R. Stickels *et al.*	Enhanced capture efficiency of the original Slide-seq.	Entire transcriptome	10 μm	Cannot be applied to FFPE tissue blocks	Commercial Curio Seeker product line: ∼$1300/sample; additional NGS required.
2021	Seq-Scope (Cho *et al.*, 2021)	Chun-Seok Cho *et al.*	Repurposing of an illumina sequencing flow cell.	Entire transcriptome	∼0.5–0.8 μm	Exposing the cluster surface of the flow cell is challenging.	∼$1500/sample; additional NGS required.
2021	Stereo-seq (Chen *et al.*, 2022)	Ao Chen *et al.*	Using DNA nanoball-patterned chips to capture RNA molecules	Entire transcriptome	∼220 nm	Low sensitivity for low abundant transcripts.	Commercial BGI Platform: ∼$1500/sample; additional NGS required.
2021	Sci-Space (Srivatsan *et al.*, 2021)	Sanjay R. Srivatsan *et al.*	Using unmodified DNA oligos to label nuclei	Entire transcriptome	Single-cell resolution	Cannot capture cytoplasmic RNA; dropout during nucleus collection.	∼$5000/sample; additional NGS required.
2022	Pixel-seq (Fu *et al.*, 2022)	Xiaona-n Fu *et al.*	Polony gel stamping enables scalable replication of DNA cluster arrays	Entire transcriptome	∼1 μm	Require specialized expertise in gel fabrication and transfer.	∼$105/sample; additional NGS required.

* Please note that this is only a rough estimate. Many of the technologies have not yet been commercialized and, therefore, the accurate information about their costs is not fully available. Furthermore, the actual cost of an experiment can vary significantly depending on experimental designs (e.g. tissue type and size, number of cells in a tissue slice, number of genes profiled, and pre-designed versus custom gene panels). Additional costs of obtaining a microscope for imaging-based technologies and next-generation sequencing for array-based technologies are not included in our calculation.

BaristaSeq: barcode *in situ* targeted sequencing; DBiT-seq: deterministic barcoding in tissue for spatial omics sequencing; FFPE: formalin fixed paraffin embedded; FISSEQ: fluorescent *in situ* sequencing; GEO-seq: geographical position sequencing; ISH: *in situ* hybridization; ISS: *in situ* sequencing; LCM: laser capture microdissection; MERFISH: multiplexed error-robust fluorescence *in situ* hybridization; NGS: next-generation sequencing; RCA: rolling circle amplification; smFISH: single-molecule fluorescence *in situ* hybridization; STARmap: spatially resolved transcript amplicon readout mapping; Stereo-seq: spatial enhanced resolution omics-sequencing.

### Targeted approaches

**Figure 1.**
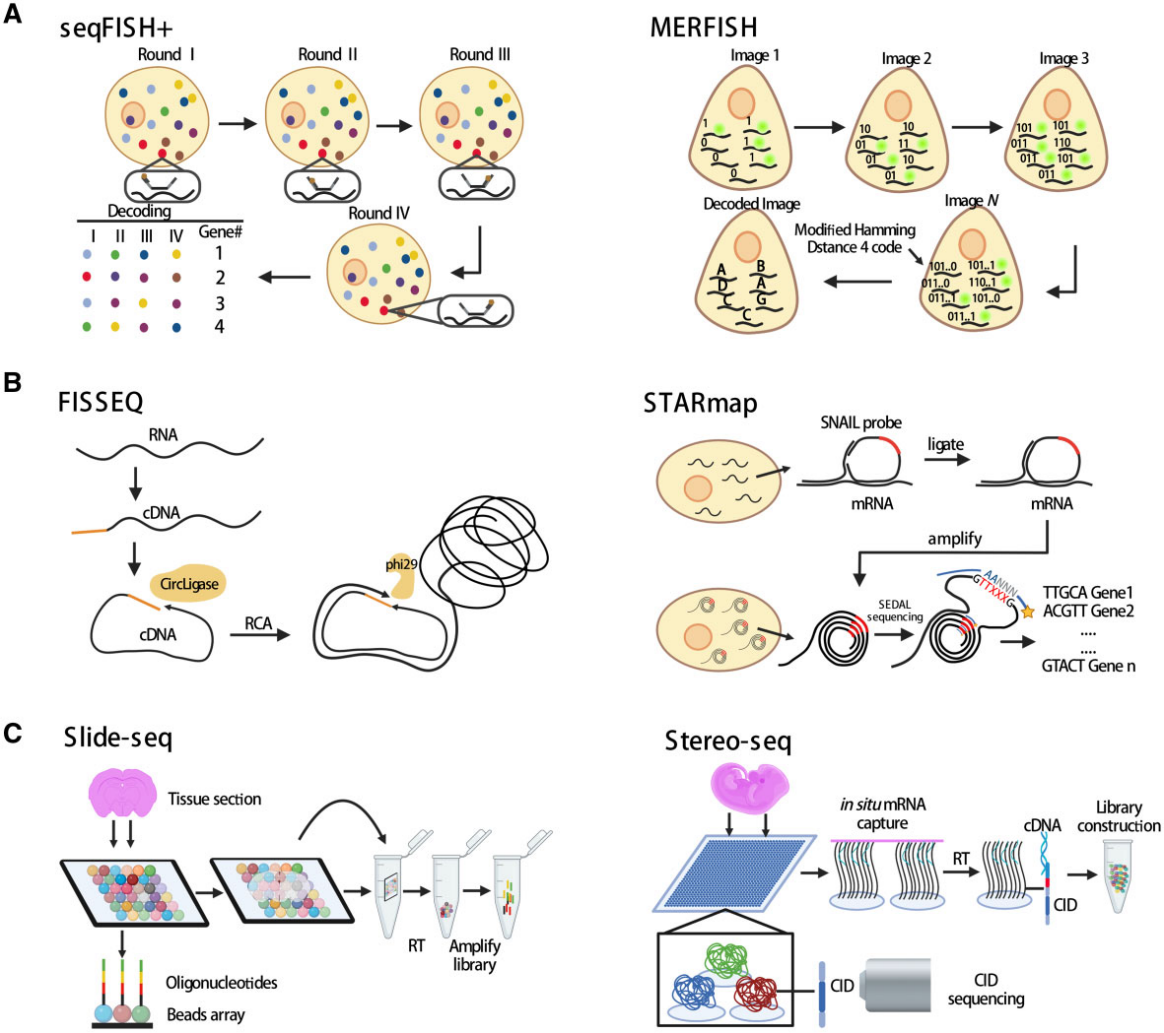
**Representative spatial transcriptomics technologies.** (**A**) Schematics of ISH-based ST technologies seqFISH+ and MERFISH. (**B**) Schematics of ISS-based ST technologies FISSEQ and STARmap. (**C**) Schematics of solid phase capture-based ST technologies Slide-seq and Stereo-seq. CID: co-ordinate identity; FISSEQ: fluorescent *in situ* sequencing; ISH: *in situ* hybridization; ISS: *in situ* sequencing; MERFISH: multiplexed error-robust fluorescence *in situ* hybridization; RCA: rolling circle amplification; RT: reverse transcription; seqFISH: sequential single-molecule fluorescence *in situ* hybridization; ST: spatial transcriptomics; STARmap: spatially resolved transcript amplicon readout mapping.

The ISH method visualizes the target molecules in tissue sections by using imaging probes to sequentially hybridize the targets. Representative techniques include single-molecule fluorescence *in situ* hybridization (smFISH) (Femino *et al.*, 1998), sequential single-molecule FISH (seqFISH) (Lubeck *et al.*, 2014), and multiplexed error-robust fluorescence *in situ* hybridization (MERFISH) (Chen *et al.*, 2015) (Fig. 1A).

In smFISH, multiple fluorescent probes target specifically complementary sites of an individual mRNA to generate high-intensity signals for visualization. This methodology accurately quantifies and visualizes the expression of RNA molecules within the cells. However, owing to a limited number of available fluorescent channels, multiple RNA targets cannot be measured simultaneously. To overcome this limitation, a technology that combines the smFISH technique with combinatorial labeling was developed. This technology, termed seqFISH (Lubeck *et al.*, 2014), decodes mRNAs by sequential rounds of hybridization, imaging, and probe stripping. Specifically, during each round of hybridization, each transcript labeled by FISH probes with a single type of fluorophore is visualized, and then, the FISH probes are removed by treatment with DNase. In a subsequent round, the same transcript is hybridized with the same FISH probes but now labeled with a different dye. Thus, four dyes and eight rounds of hybridization can theoretically cover the entire transcriptome of the mouse or the human (4^8^ = 65 536). However, global profiling of hundreds or thousands of mRNA is hindered by optical crowding. To overcome this challenge, seqFISH+ was developed (Eng *et al.*, 2019). seqFISH+ expands the barcode base palette from 4 to 5 colors to 60 pseudocolors per image cycle, resulting in the detection of ∼10 000 genes per cell by repeating the cycle of pseudocolor imaging only four times (Fig. 1A, left).

Another ISH-based method is MERFISH (Chen *et al.*, 2015), which uses the modified Hamming code to encode the RNA molecules (Fig. 1A, right). Using a two-step labeling scheme, MERFISH dramatically decreases the probe hybridization time and reduces the error rate of barcode identification. Furthermore, the detection efficiency of RNA molecules by MERFISH can be increased through a combination with expansion microscopy, which effectively increases the distances between neighboring RNA molecules and helps substantially increase the RNA density measurable by MERFISH (Wang *et al.*, 2018c). Thus, the gene throughput of MERFISH has been increased from the original ∼1000 transcripts to ∼10 000 transcripts in individual cells (Xia *et al.*, 2019).

Besides ISH-based approaches, ISS-based methods are also frequently used to yield spatial transcriptome information (Fig. 1B). In 2013, an ISS technology that combines padlock probing, rolling circle amplification (RCA), and sequencing-by-ligation chemistry was used to sequence RNAs *in situ* for the first time (Ke *et al.*, 2013). In ISS, the padlock probes that carry transcript-specific barcodes hybridize to the RNA targets and are circularized via ligation of the 5′ and 3′ ends of the probes. Then, the circularized padlock probes are amplified by RCA and the probe barcodes are sequenced *in situ* using fluorescent oligos. Similarly, fluorescent *in situ* sequencing (FISSEQ) (Lee *et al.*, 2014), another ISS-based method, first generates cDNA from RNA using reverse transcription with tagged random hexamers. Then, the cDNA fragments are circularized by circligase and amplicons are formed after RCA. This procedure ensures that RNA molecules are profiled in a non-targeted manner. Spatially resolved transcript amplicon readout mapping (STARmap) is another technology based on ISS (Wang *et al.*, 2018a). It uses a pair of primer and padlock probes (called SNAIL probes) to ensure target-specific signal amplification. STARmap bypasses the step of reverse transcription to increase the efficiency of amplicon generation. In addition, an improved ISS chemistry called SEDAL was devised specifically for STARmap. SEDAL eliminates error accumulation as sequencing proceeds and exhibits minimal background. With these improvements, STARmap reads >1000 genes per cell in a mouse brain.

### Unbiased approaches

In the early days of ST, physical microdissection techniques were used to isolate molecules at specific spatial locations, such as those in LCM (Emmert-Buck *et al.*, 1996), Tomo-Seq (Junker *et al.*, 2014), and spatial transcriptomics by reoriented projections and sequencing (STRP-seq) (Schede *et al.*, 2021). LCM can efficiently and accurately obtain target cell subgroups or single cells within tissues (Emmert-Buck *et al.*, 1996), and is often used to analyze the transcriptome of tissue regions in combination with other sequencing methods. For example, geographical position sequencing (Geo-seq) captures cell heterogeneities and spatial variance simultaneously by combining LCM with single-cell RNA sequencing (scRNA-seq) technology (Chen *et al.*, 2017). Similarly, LCM-seq combines LCM with poly A-based Smart-seq2 RNA sequencing (Nichterwitz *et al.*, 2016, 2018).

Although LCM combined with scRNA-seq can provide ST information at cellular resolution, its low throughput makes it difficult to scale to large tissue areas. To overcome this limitation, a solid-phase capture technology named Spatial Transcriptomics was developed in 2016 (Ståhl *et al.*, 2016). Its innovation lies in the introduction of spatial barcodes before sequencing library preparation (Jemt *et al.*, 2016). Specifically, the mRNA molecules of tissue sections are captured with spatially barcoded oligo(dT) primers anchored on glass slides. The subsequent reverse transcription enables the resulting cDNAs to be coupled to the arrayed oligo(dT) primers on the glass slides. By using NGS, the mRNA identity and the coupled spatial barcode can be identified. Each gene can then be unbiasedly mapped to the tissue sections based on the unique spatial barcode. Thus, Spatial Transcriptomics quantifies the gene expression and visualizes the distribution of mRNAs within tissue sections. The spatial resolution of Spatail Transcriptomics is 100 μm with a center-to-center distance of 200 μm between two adjacent ‘spots’. Building upon the ST technology, the commercially available 10× Genomics Visium technology (10× Genomics, 2019) increases the cellular resolution to 55 μm with a 100-μm center-to-center distance between spots and a sensitivity of >10 000 transcripts per spot.

Slide-seq is another ST technology that combines spatial barcoding with solid-phase RNA capture (Rodriques *et al.*, 2019) (Fig. 1C). The Slide-seq array is generated by packing DNA-barcoded beads onto a glass surface. The position of each bead is determined by ISS. Using the spatially indexed arrays, Slide-seq captures mRNA molecules from fresh frozen tissue sections and enables unbiased mapping of the mRNA molecules back to the original locations. Compared with Spatial Transcriptomics and 10× Genomics Visium, Slide-seq provides a higher spatial resolution (10-μm bead diameter) and lower experimental cost. Slide-seqV2 (Stickels *et al.*, 2021), an improved version of Slide-seq, has an RNA capture efficiency of ∼10-fold greater than the original Slide-seq, resulting from the improved workflow of library generation, bead synthesis, and array indexing.

Recently, spatial enhanced resolution omics-sequencing (Stereo-seq) has achieved nanoscale resolution (220-nm spot diameter with ∼500-nm center-to-center distance) by using spatially barcoded DNA nanoball (DNB) chips (Fig. 1C). The spatial location of each DNB can be read out by sequencing. The high sensitivity and resolution allow Stereo-seq to be used to visualize nuclear versus cytoplasmic transcripts. Other examples of ST technologies based on solid-phase capture and spatial barcoding include deterministic barcoding in tissue for spatial omics sequencing (DBiT-seq) (Liu *et al.*, 2020), sci-Space (Srivatsan *et al.*, 2021), and Pixel-seq (Fu *et al.*, 2022).

## Biological insights of mammalian reproduction revealed by ST technologies

ST technologies have been widely applied for the visualization of molecular spatial structures within various tissues (Garcia-Alonso *et al.*, 2022; Guilliams *et al.*, 2022; Hwang *et al.*, 2022; Kuppe *et al.*, 2022; Ratz *et al.*, 2022). By capturing the spatial context of RNA molecules, ST complements scRNA-seq for biological discoveries. In the following sections, we systematically review the novel biological insights of mammalian reproduction revealed by ST technologies, including the identification of genes with non-random spatial expression patterns and specialized functions; the characterization of cellular neighborhoods under reproductive homeostasis; and the examination of tissue microenvironment under pathological conditions.

### Spatially patterned gene expression and functions in reproductive systems

Genes with non-random spatial distributions within a tissue often play important roles in cellular functions. To this end, ST technologies offer a unique opportunity to identify these genes at scale.

**Figure 2.**
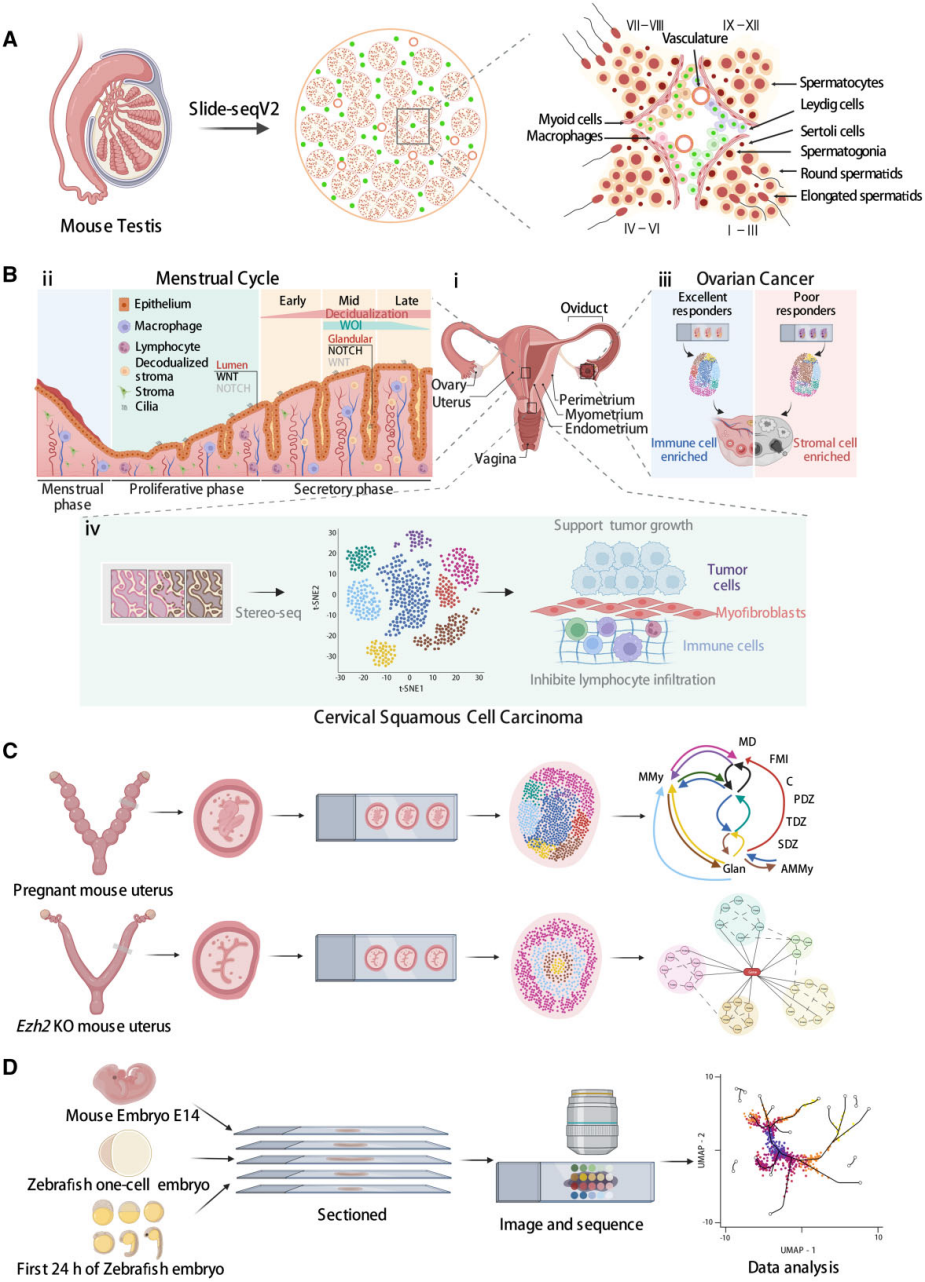
**Applications of spatial transcriptomics technologies in reproductive systems.** (**A**) The establishment of an unbiased spatial transcriptome atlas of mammalian spermatogenesis using Slide-seqV2. (**B**) (i) An overview of the human female reproductive system. (ii) The cellular structure and molecular signaling of the human endometrium throughout the menstrual cycle. (iii) Spatial characterization of high-grade serous ovarian carcinoma tumor tissue from poor and excellent responders to neoadjuvant chemotherapy. (iv) Stereo-seq identifies cancer-associated myofibroblasts, which may play a supporting role in tumor growth and metastasis by inhibiting lymphocyte infiltration and remodeling tumor extracellular matrix in cervical squamous cell carcinoma. (**C**) Upper panel: spatial cellular neighborhoods of the mouse uterus at the embryo implantation site. AMMy: anti-mesometrial myometrium; E: embryo; FMI: fetal–maternal interface; Glan: uterine glands; MD: mesometrial decidua; MMy: mesometrial myometrium; PDZ: primary decidual zone; SDZ: secondary decidual zone; TDZ: transition decidual zone. Lower panel: the spatial gene expression profile of the enhancer of zeste homolog 2 (*Ezh2*) knockout (KO) mouse uterus. (**D**) Dissecting the developmental processes of mouse and zebrafish embryos using spatial transcriptomics technologies.

For example, seminiferous tubules are the functional units of spermatogenesis in mammalian testes (Hess and Renato de Franca, 2008) (Fig. 2A). In a recent study, Slide-seqV2 was used to capture the spatial distribution of testicular genes in the mouse and human testis at a high throughput (Chen *et al.*, 2021b). Computational analysis of the Slide-seqV2 data systematically revealed genes with non-random spatial distribution in seminiferous tubules such as genes enriched at the periphery of a tubule versus genes enriched near the center of a tubule. The analysis also identified genes whose expression is restricted to a subset of seminiferous tubules. Among these genes, *Habp4* (hyaluronan binding protein 4) was discovered as a potential novel regulator of the chromatin remodeling process during male germ cell development. Furthermore, by comparing the gene expression profiles of Leydig cells (the testosterone-producing somatic cells in the interstitial space), the authors showed that Leydig cells that are spatially adjacent to a subset of seminiferous tubules express a high level of *1700017N19Rik*. These *1700017N19Rik*-expressing Leydig cells also express the stem Leydig cell marker *Nr2f2* (nuclear receptor subfamily 2 group F member 2), indicating that *1700017N19Rik* may be involved in the regulation of stem Leydig cell functions. A similar analysis was performed on testicular macrophages, which identified two spatially distinct macrophage subpopulations. One population localizes in the interstitial space and the other is enriched in the peritubular space. These two populations can be distinguished by the expression of *H2-Ab1* (histocompatibility 2, class II antigen A, beta 1) and *Il1b* genes that are exclusively expressed in peritubular macrophages. Besides Leydig cells and macrophages, it is likely that other testicular somatic cells, such as Sertoli cells and myoid cells, also exhibit spatially dependent gene expression patterns. However, the spindle shape of the myoid cells as well as the spatial proximity of Sertoli cell cytoplasm and that of germ cells make it challenging to capture the myoid cell-specific or Sertoli cell-specific transcriptome using Slide-seqV2. This is because one spot on the Slide-seqV2 array may capture mRNA transcripts from two to three adjacent cells. Thus, single-cell-level ST approaches may be better at resolving the spatial transcriptome in cells with irregular shapes or small sizes. For example, seqFISH, a single-cell ST approach, was employed to probe the spatial expression patterns of marker genes of human spermatogonial sub-states identified by scRNA-seq (Guo *et al.*, 2018). This analysis showed that *PIWIL4* (piwi-like RNA-mediated gene silencing 4) and *ETV5* (ETS variant transcription factor 5)/*L1TD1* (LINE1-type transposase domain containing 1) are enriched in spatially distinct spermatogonium (SPG) subpopulations.

The human endometrium is another example in which ST technologies have been employed to reveal spatial gene expression pattens (Fig. 2B, ii). Studying human endometrial homeostasis and pathology has been challenging owing to a lack of model systems (Maurya *et al.*, 2021). One study combined scRNA-seq with ISH to characterize the human endometrium across the menstrual cycle (Wang *et al.*, 2020). Besides the canonical cell types such as stromal fibroblast, endothelium, macrophage, and lymphocyte, scRNA-seq analysis also identified an epithelium-associated cell type the authors called ‘ciliated epithelium’. Four genes were found to be highly discriminatory for these ciliated cells (*C11orf88*, *C20orf85*, *FAM183A* (family with sequence similarity 183 member A), and *CDHR3* (cadherin-related family member 3)). ISH targeting of these four genes revealed their consistent co-expression with FOXJ1 protein (forkhead box J1, a master regulator for motile cilia with epithelial lineage identity) in both glandular and luminal epithelia on Days 17 and 25 of the menstrual cycle. In another study, Garcia-Alonso *et al.* (2021) examined the human endometrial epithelium using 10× Genomics Visium. They spatially resolved five cell clusters corresponding to cells in the luminal, functional, and basal layers. Gene signatures of WNT and NOTCH signaling pathways were found to be present in distinct endometrial locations. For example, genes encoding the WNT pathway components, *FOXJ1* and *LGR5* (leucine-rich repeat-containing G protein-coupled receptor 5), are enriched at the luminal surface while *NOTCH2* (notch receptor 2) is mainly expressed in glands in the functional layer. Furthermore, the authors found that *NOTCH2* expression increases in glands moving away from the lumen while *WNT7A* (wnt family member 7A) expression is higher in the luminal epithelium compared with glands. By contrast, the noncanonical WNT gene *WNT5A* is enriched in stromal cells surrounding the glands. These findings suggest an almost mutually exclusive spatial expression pattern between the canonical and noncanonical WNT pathways in the glandular microenvironment.

Besides the human, ST technologies have also been applied to study the mouse uterus in a uterine *Ezh2* (enhancer of zeste homolog 2) knockout (KO) model (Mesa *et al.*, 2021) (Fig. 2C, lower panel). EZH2 is an epigenetic modifier that methylates histone lysine residue 27 (Trevino *et al.*, 2015). Conditional deletion of *Ezh2* in the uterus results in an increased proliferation of luminal and glandular epithelial cells and affects the estrogen signaling pathway (Fang *et al.*, 2019; Nanjappa *et al.*, 2019). Spatial analysis of the uterine *Ezh2* KO model versus the wild type (WT) using 10× Genomics Visium allowed a specific selection of epithelial cells for downstream analyses. Differential expression analysis identified up-regulated (*Asb4* (ankyrin repeat and SOCS box-containing 4), *Cxcl14* (chemokine (C-X-C motif) ligand 14), *Dio2* (deiodinase, iodothyronine, type II), and *Igfbp5* (insulin-like growth factor-binding protein 5)) and down-regulated (*Sult1d1* (sulfotransferase family 1D, member 1), *Mt3* (metallothionein 3), and *Lcn2* (lipocalin 2)) genes in *Ezh2* KO versus WT uterine epithelium.

Finally, multiple ST technologies have been used to spatially profile gene expression during embryogenesis (Fig. 2D). In one study, spatial analysis of mouse E14.0 embryos using sci-Space revealed spatially patterned, cell-type-specific gene expression across the embryo (Srivatsan *et al.*, 2021). Follow-up analyses distinguished genes whose spatial pattern of expression is contributed by multiple cell subtypes from genes whose spatial pattern of expression is contributed by the presence of a single spatially restricted, unannotated cell subtype. For example, the spatial expression pattern of *Hox* genes, a class of homeotic transcription factors that specify the body plan, could not be explained solely by spatial restriction of a single-cell subtype. The spatial expression of *Cyp26b1*, a gene encoding a member of the cytochrome P450 superfamily, is restricted to the brainstem with expression observed in multiple neuronal subclusters. In another study, LCM-based Tomo-seq was applied to zebrafish embryos (Holler *et al.*, 2021). The authors sorted genes based on their spatial expression patterns along the animal-to-vegetal axis of the embryo. As a result, three major groups of spatially patterned genes were identified. One group localizes to the animal side of the embryo, one group of genes is equally distributed across all sections, and a third group of genes is spatially confined to the most vegetal part of the yolk sac. By combining Tomo-seq data of *Xenopus laevis* and *Xenopus tropicalis* embryos with that of the zebrafish embryos, the authors identified nine genes, such as *dazl* (deleted in azoospermia like) and *camk2g1* (calcium/calmodulin-dependent protein kinase II gamma 1), that localize vegetally in all three species, suggesting their conserved function in germ cell development or dorsoventral axis development.

### Cellular neighborhoods and their functional implications in reproductive physiology

Another key feature of ST is the ability to identify spatial clustering of interacting cell populations (i.e. cell neighborhoods) within the tissue context.

In the testis, developing gametes are regulated by the tissue microenvironment consisting of various somatic cell types (Sertoli cells, Leydig cells, myoid cells, endothelial cells, macrophages, etc.) (Phillips *et al.*, 2010; Wu *et al.*, 2020) (Fig. 2A). Therefore, understanding the interplay between the developing gametes and the somatic cells is essential to the understanding of spermatogenesis. Using the Slide-seqV2 data, one study calculated the cellular compositions of the tissue microenvironment surrounding SPG (the stem cell-containing developing gametes) by identifying their cellular neighborhoods (Chen *et al.*, 2021b). It was found that in both the mouse and human testis, undifferentiated and differentiating SPG self-aggregate while also spatially segregating from each other. Furthermore, no difference in the spatial compositions of the microenvironment surrounding the undifferentiated versus differentiating mouse SPG was found, which was further validated by an independent ISS experiment targeting 22 testicular marker genes. Of interest, in contrast to the mouse, significant differences in the spatial cellular compositions of the microenvironment surrounding the human undifferentiated versus differentiating SPG were identified. For example, a differential enrichment of endothelial cells in the microenvironment surrounding the human undifferentiated versus differentiating SPG was noted. Together, this study revealed differences in the spatial structure of the spermatogonial microenvironment between the mouse and the human, indicating differential regulatory mechanisms governing the early stage of spermatogenesis between the two species.

During pregnancy, the relationship between the placenta and the decidua of the uterus is essential to nurture and protect the fetus. To this end, one study focused on the development of trophoblast cells at the human maternal–fetal interface by integrating single-nucleus RNA sequencing (snRNA-seq) with 10× Genomics Visium (Arutyunyan *et al.*, 2023). Different trophoblast subsets were annotated and spatially mapped in the tissue. These trophoblast cells were further grouped into five pre-defined microenvironments in the tissue based on histological features, and distinct trophoblast subsets were found in different microenvironments. Furthermore, by ordering trophoblast states based on their proximity in both the gene expression and physical space, the most likely trajectory for the emergence and differentiation of invasive extravillous trophoblast cells (EVTs) was inferred (Arutyunyan *et al.*, 2023). This analysis showed that a subset of EVTs (EVTs-2) can transit either into interstitial EVTs that invade through decidual stroma or into endovascular EVTs that move down inside the arteries. Thus, this study demonstrates how high-quality single-cell and spatial data can be integrated to identify the spatial organization of cell-type subsets and their developmental relationships.

Similarly, normal uterine functions are required during pregnancy and depend on crosstalk among multiple cell types in uterine microenvironments. By applying 10× Genomics Visium to the embryo implantation site of the mouse uterus on pregnancy Day 7.5, Li *et al.* (2022) identified 11 cellular neighborhoods, including a mesometrial myometrium (MMy), an anti-mesometrial myometrium, a mesometrial decidua (MD) enriched with natural killer (NK) cells, a vascular sinus zone (VSZ) for maternal vessel remodeling, a fetal–maternal interface (FMI), a primary decidual zone (PDZ), a transition decidual zone (TDZ), a secondary decidual zone (SDZ), undifferentiated stroma (udStr), uterine glands, and the embryo (Fig. 2C, upper panel). Consistent with previous histological studies, the authors found that the MMy is located next to the mesometrial uterine artery, and the embryo environment is surrounded by the decidual zone composed of polyploid decidual cells. Analysis of gene expression further showed that the decidual zone can be divided into the PDZ, the SDZ, and the TDZ. The PDZ consists of cells that express high levels of prolactin genes *Prl3c1*, *Prl8a2*, and troponin gene *Tnnc1*. In contrast, the SDZ expresses metallothionein genes *Mt3*, *Mt4*, and cochlin. Using the ST data, the authors identified three major communication regions among the uterine neighborhoods. The first one consists of the embryo and its adjacent FMI and PDZ microenvironments. The other two regions can be divided into the mesometrial pole and the anti-mesometrial pole. The mesometrial pole is made up of the MD, VSZ, and FMI, while the anti-mesometrial pole consists of PDZ, TDZ, SDZ, and udStr. These neighborhoods communicate not only within the regions but also between regions. Together, this study demonstrates the complex molecular and cellular interactions that occur during early pregnancy. Given the low spatial resolution of the 10× Genomics Visium technology, future studies using single-cell-level ST approaches may better resolve the spatial heterogeneity of cell-type distributions and communications in the uterine microenvironment.

Besides the testis and the uterus, the mouse placenta has also been studied using ST to reveal its cellular neighborhoods. By using STARmap to target 903 placental genes, He *et al.* (2021) discovered distinct spatial patterns of placental cell types. The authors found that a subset of maternal decidua cells (MD-1), a subset of trophoblast giant cells (TG-2), and maternal NK cells mainly self-aggregate, while a subset of glandular trophoblast cells (GT-2), TG-1, TG-3, endothelial, and stromal cells exhibit high spatial mixing with each other. Furthermore, to investigate if the neighbors of a cell influences the gene expression of the cell, the authors performed clustering of MD-1 cells based on their gene expression and cellular neighborhood compositions, respectively. Both clustering results identified the same two subtypes, suggesting that the spatial environment may shape the gene expression landscape of MD-1 cells. Future functional studies are needed to go beyond this correlation analysis to establish causality between cellular microenvironment and gene expression.

Finally, ST technologies have also been applied to embryos for tissue structure analysis (Fig. 2D). For example, Chen *et al.* (2022) applied Stereo-seq to mouse embryos. In the spinal cord region of an E13.5 embryo, the authors identified the *Hopx* (HOP homeobox)^+^ ventricular zone, *Slc5a7* (solute carrier family 5, member 7)^+^ marginal zone, *Vsnl1* (visinin-like 1)^+^ basal plate, *Fut9* (fucosyltransferase 9)^+^ ventral and *Hoxb8*^+^ lateral parts of the spinal alar plate, and *Pdyn* (prodynorphin)^+^ superficial stratum of spinal basal plate. Furthermore, spatial clustering of cell types in the embryonic brain recapitulated known anatomically defined brain regions including the ventricular and mantle zones of the pallium, subpallium, midbrain, hindbrain, diencephalon, cerebellum, hypothalamus, olfactory bulb, and choroid plexus. In another study, Srivatsan *et al.* (2021) used sci-Space data of mouse embryos to delineate the spatial gradients of cellular differentiation and neuronal migration. The authors found that in the pallium, immature neurons migrate and differentiate radially outward, leading to the inside-out development of the cortical layers. In the subpallium, cortical interneurons born in the ganglionic eminences migrate tangentially to populate the developing cortex and olfactory bulb. Moreover, midbrain neurons seem to migrate both radially, toward the pial surface, and tangentially, parallel to the pial surface, to populate this region. Together, these studies demonstrate the ability of ST technologies to resolve complex tissue structure, such as the embryo.

### Reproductive pathology-associated spatial microenvironments

Pathological states in a tissue are often associated with altered cellular microenvironments. By identifying genes with spatially patterned expressions and spatially clustered cell populations, ST is well positioned to detect and characterize such alterations.

Diabetes mellitus has been known to impact male fertility through multiple mechanisms, such as disruption of spermatogenesis, testicular degeneration and apoptotic changes, and endocrine disorders (Bhat *et al.*, 2006; Agbaje *et al.*, 2007; Ricci *et al.*, 2009; Schoeller *et al.*, 2012; Jangir and Jain, 2014; Maresch *et al.*, 2018). Applying Slide-seqV2 to testis samples from leptin-deficient diabetic mice (*ob/ob*) and WT mice identified genes with altered spatial expression patterns such as *Smcp* (sperm mitochondria-associated cysteine-rich protein) and *Malat1* (metastasis-associated lung adenocarcinoma transcript 1). Further analysis of Slide-seqV2 data showed a significant increase in the extent of spatial mixing between haploid spermatids and other testicular cell types in *ob/ob* seminiferous tubules (Chen *et al.*, 2021b), suggesting that the disruption of the spatial structure of seminiferous tubules is a potential mechanism of diabetes-induced testicular injuries.

Besides the testis, other male reproductive organs, such as the prostate, have also been investigated using ST approaches. Hirz *et al.* (2023) applied Slide-seqV2 to study the prostate tumor microenvironment. First, compared to the spatial configuration of the healthy prostate tissue in which well-organized prostate epithelial glands are surrounded by immune and non-immune stromal cells including fibroblasts, pericytes, and endothelial cells, the tissue architecture was notably disrupted in the cancerous prostate. The authors found that the spatial distributions of fibroblasts, endothelial cells, and pericytes became more dispersed compared to those in healthy tissues. Second, compared to the healthy prostate tissue in which an organized glandular epithelium contains a well-structured bilayer of basal and luminal cells, there was an expansion of the luminal epithelial population and loss of the well-organized glands in tumor-adjacent normal sample. Third, among the four epithelial subpopulations, the spatial organizations of the club and hillock cells were disrupted in the tumor and tumor-adjacent normal tissues. Finally, to infer cell–cell communications (CCC), the Slide-seqV2 data were used to construct a graph of physically adjacent cells, which permitted testing of whether a ligand–receptor (LR) score, defined as a product of the two corresponding expression levels, was significantly higher in physically adjacent cells than would be expected from a randomized spatial arrangement. This analysis revealed 405 statistically significant potential LR interactions. Focusing on tumor–stromal communication, the authors found that tumor cells expressing vascular endothelial growth factors (VEGFA and VEGFB) can stimulate a subpopulation of endothelial cells through VEGF receptors, FLT143 and beta-1 integrin. Potential interactions between tumor cells and fibroblasts (COL9A2-ITGA1) and tumor cells with a subpopulation of pericytes cells (COL12A1-ITGA1) were also identified. Together, this study demonstrates the power of ST in dissecting the prostate tumor microenvironment as well as tumor–stromal cell interactions.

In the human uterus, Garcia-Alonso *et al.*, identified four main groups of human endometrial epithelial cells based on their marker gene expression using scRNA-seq: a *SOX9* (SRY-box transcription factor 9)^+^ population; *PIFO* (primary cilia formation)^+^*TPPP3* (tubulin polymerization promoting protein family member 3)^+^ ciliated cells; *LGR5*^+^ lumenal cells; and *SCGB2A2* (secretoglobin family 2A member 2)^+^ glandular cells. Further analysis of the *SOX9*^+^ population revealed three cell clusters: *SOX9*^+^*LGR5*^+^ cells; *SOX9*^+^*LGR5*^−^ cells; and proliferative *SOX9*^+^ cells. By integrating scRNA-seq and 10× Genomics Visium data, the authors showed that *SOX9*^+^*LGR5*^+^ cells are spatially enriched in the surface epithelium; *SOX9*^+^*LGR5*^−^ cells locate in the basal glands; and proliferative *SOX9*^+^ cells are spatially mapped to glands in the regenerating superficial layer. ISH experiments further confirmed the spatial distribution of proliferative *SOX9*^+^ cells by showing high expression of the proliferative marker *MKI67* in the superficial layer of the endometrium during the proliferative phase (Garcia-Alonso *et al.*, 2021). In the same study, the authors correlated the clinical stages of endometrial adenocarcinomas with the three clusters of the *SOX9*^+^ population. The more advanced stages of endometrial adenocarcinomas (stages III and IV) were found to have a greater *SOX9*^+^LGR5^+^ signal. This *SOX9*^+^*LGR5*^+^ signal is also stronger in endometrial tumors characterized by high copy number alterations and is linked with a worse prognosis (Garcia-Alonso *et al.*, 2021). These data demonstrate the importance of pinpointing the molecular and spatial identity of cellular subtypes for disease diagnosis and treatment. In another study, Fonseca *et al.* (2023) applied 10× Genomics Visium to study endometriosis—a disease characterized by endometrial-like tissue growing outside of the uterine cavity. *FOXJ1*^+^ ciliated cells and *LGR5*^+^ and *SOX9*^+^ cells were found to be surrounded by *KRT10* (keratin 10)^+^ cells both within and outside of the endometriosis lesion proper. Furthermore, *ECM1* (extracellular matrix protein 1)^+^ and *MMP11* (matrix metallopeptidase 11)^+^ endometrial-type stroma cells were detected in the endometriosis lesions. *CFD* (complement factor D)^+^ peritoneal fibroblasts were separated from the lesions by a region of *C7* (complement C7)^+^ fibroblasts scattered with *FAP* (fibroblast activation protein alpha)^+^ cells. This study demonstrates the cellular and spatial heterogeneity in the endometriosis lesion.

In the ovary, high-grade serous ovarian carcinoma (HGSC) is the most common type of ovarian cancer and is also highly chemosensitive. Platinum-based combination chemotherapy is an important treatment for this disease, but patient responses to the treatment vary significantly (Matulonis *et al.*, 2016). The mechanisms behind this diversity in response to treatment are unclear (Peres *et al.*, 2020). Stur *et al.* (2022) applied 10× Genomics Visium to investigate the reasons behind the different responses to neoadjuvant chemotherapy from patients with HGSC. The authors uncovered more stromal-dominated cell groups, largely formed by myofibroblasts rather than conventional cancer-associated fibroblasts in the tumor samples from the poor responder (PR) group. By contrast, tumors of excellent responders (ER) contain a high proportion of immune cells, including T cells, B cells, and NK cells. Unsupervised clustering of the ST data revealed nine cell clusters. Significant differences in the spatial distribution of these clusters were observed. For example, in the PR group, the clusters are physically larger and distributed throughout the whole tissue area, whereas in the ER group, clusters are smaller and more compact. Furthermore, some clusters are located close to each other in one group but significantly farther from each other in the other group, indicating differential cell-to-cell contacts in different groups (Fig. 2B, iii). Follow-up studies on how the cell-to-cell contacts differ in the two patient groups at the molecular level (e.g. LR interactions) would provide mechanistic insights into the differential responses to chemotherapy from patients with HGSC.

In addition to ovarian cancer, cervical cancer also threatens the reproductive health of women worldwide. Recently, Ou *et al.* (2022) used snRNA-seq and Stereo-seq to analyze the gene expression patterns and cellular interactions in cervical squamous cell carcinoma tumors (Fig. 2B, iv). The authors identified six tissue clusters based on gene expression patterns: tumor, stroma (without obvious inflammation), inflammation (stroma with diffuse inflammation or focal inflammation), gland, blood vessel, and necrosis. The tumor cluster was further divided into hypermetabolic tumor and hypometabolic tumor based on the expression level of genes associated with oxygen status and energy production pathways. Of interest, a unique spatial cluster largely composed of cancer-associated myofibroblasts (myCAFs) was found outside the hypermetabolic tumor regions. Differential gene expression analysis showed that the myCAF^+^ tumors are more active in energy usage, metabolism, mitosis, and cell growth than myCAF^–^ tumors, whereas signaling activities associated with cellular adhesion, apoptosis, and immune responses are down-regulated in myCAF^+^ tumors. These observations indicate that the presence of myCAFs may play important roles in supporting cervical cancer progression.

## Challenges and outlook of applying ST technologies to studying mammalian reproduction

A growing number of studies, as reviewed above, have demonstrated the utility of ST technologies in revealing the biological regulation of reproductive physiology and pathology. However, challenges remain to apply ST technologies to study reproductive systems.

### Experimental challenges

The number of unique molecules per cell captured by the current ST technologies is, in most cases, less than that captured by the state-of-art scRNA-seq technologies. This has hindered the spatial profiling of lowly expressed genes and rare cell types in reproductive systems. At the RNA level, the current ST methods mostly focus on the detection of mRNAs, whereas the spatial information of non-coding RNAs is rarely resolved, even though non-coding RNAs play important roles in reproductive systems (Bourc'his and Voinnet, 2010; Cabili *et al.*, 2011; Pauli *et al.*, 2011; McIver *et al.*, 2012; de Mateo and Sassone-Corsi, 2014). A combination of *in situ* RNA polyadenylation with existing ST technologies may solve the issue (McKellar *et al.*, 2022). Furthermore, many ST technologies are optimized for fresh frozen tissue specimens and have a high requirement for RNA integrity, preventing their applications in clinical research where clinical specimens are often formalin fixed and paraffin embedded and contain a large quantity of fragmented RNAs. Thus, for samples with low RNA quality, a targeted gene panel may be applied to enhance the capture efficiency of the ST technologies (Mirzazadeh *et al.*, 2023). Finally, many ST technologies rely on specialized equipment or custom-made arrays. Although several commercial ST solutions are available, the high costs of these solutions limit their accessibility to non-specialist laboratories (Table 1). Institutional or regional core facilities that provide these commercial solutions on a fee-for-service basis would help to democratize the use of ST technologies.

### Computational challenges

Current ST approaches span a wide range of spatial resolution, from broad tissue regions to subcellular localization (Table 1). In reproductive systems, cell sizes vary significantly among different cell types and even within cells belonging to the same cell type but at different developmental stages. The ability to accurately perform cellular segmentation on the measured molecules is, therefore, crucial to many downstream applications, such as quantifying cell-type composition and tissue organization. For example, in ISH- and ISS-based ST approaches, individual transcripts need to be grouped into cells from microscopy images based on image masks generated by a segmentation algorithm. This algorithm often needs extensive customization and fine tuning for each tissue type. Thus, innovations in computer vision, such as the recent machine learning-based approaches (Berg *et al.*, 2019; Pachitariu and Stringer, 2022), will greatly accelerate the ability of ST tools to be applied to various reproductive organs.

Another key computational challenge is to analyze CCC specifically for ST data. While many analyses focus on the structural relationship of cells, such as calculating the frequencies or pairwise co-occurrence of cell types in different tissue regions, few tools are available to model CCC at the molecular level. Current methods to examine molecular CCC do so in a pairwise and local manner, focusing on information between cells or in the neighborhoods of individual cells (Cang and Nie, 2020; Dries *et al.*, 2021; Garcia-Alonso *et al.*, 2021; Shao *et al.*, 2022). As a result, the collective or global information in CCC, such as the competition between cells, and long-range cell–cell interactions, such as the endocrine and telecrine signaling (both are common regulatory mechanisms in the reproductive organs), are neglected. Incorporating prior knowledge of cell–cell competition and classification of LR interactions into short-range and long-range communications might be helpful to infer the comprehensive communication categories computationally.

### The outlook

The rapid progress in the development of ST technologies will open new possibilities for the study of reproductive systems and beyond. We anticipate several exciting new directions the field is heading.

First, going beyond capturing a snapshot of molecular abundancy in a spatially resolved manner, ST technologies can be applied to measure cellular dynamics. For example, by combining ethynyl-2′-deoxyuridine labeling of the transcriptome with STARmap, temporally resolved *in situ* sequencing and mapping was recently developed to simultaneously profile the age and location of individual RNA molecules within intact cells and tissues (Ren *et al.*, 2023). Furthermore, novel temporal recording technologies have enabled the encoding of cellular lineages (Shipman *et al.*, 2016; Frieda *et al.*, 2017; Chen *et al.*, 2020; Choi *et al.*, 2022) and transcriptomic states (Chen *et al.*, 2021a; Rodriques *et al.*, 2021) in the form of DNA or RNA mutations. Combining these recording approaches with ST technologies may reveal cellular histories and dynamics during gamete development and embryonic development within the native tissue context.

Second, progress in single-cell technologies has already enabled multi-modal profiling of the transcriptome, the proteome, and the epigenome (Zhu *et al.*, 2020; Ogbeide *et al.*, 2022). Spatial multi-omics technologies may provide solutions for spatially resolved muti-modal profiling. Recent developments in DNA-tagged antibodies and application of LCM have enabled highly multiplexed protein or whole proteome readouts (Goltsev *et al.*, 2018; Merritt *et al.*, 2020; Mund *et al.*, 2022), respectively. The protein A-Tn5 transposase fusion has enabled highly multiplexed spatial readouts of the epigenome (Deng *et al.*, 2022a,b; Lu *et al.*, 2022). These approaches can be readily coupled with ST measurements. For instance, spatially resolved co-capture of the transcriptome and the epigenome in E13 mouse embryo has been proven feasible (Zhang *et al.*, 2023). Soon, whole proteome-targeting antibody/nanobody libraries may be developed for *in situ* measurements.

Finally, ST technologies may offer an opportunity to dissect gene functions at scale within the native tissue context. For biological processes like gametogenesis, thousands of genes are involved, which makes it difficult to pinpoint the functional contribution of each gene. Traditionally, the *in vivo* functions of a gene can be analyzed by generating KO mouse lines. However, this approach demands significant time and resources, making it challenging to scale. Emerging technologies, such as clustered regularly interspaced short palindromic repeats (CRISPR) screens coupled with scRNA-seq, can examine gene functions at scale (Dixit *et al.*, 2016; Datlinger *et al.*, 2017). While cell-intrinsic effects of a gene perturbation may be read out using scRNA-seq, the extracellular effects of a gene perturbation cannot be assessed owing to tissue disassociation. This excludes using CRISPR screens to identify genes controlling phenotypes that require spatial resolution to assess, such as genes encoding secreted factors. Therefore, future efforts to develop a CRISPR screen approach that retains the spatial context of a biological process will enable profiling of phenotypes that cannot be accessed in the absence of tissue context, such as cellular localization and cell–cell interactions.

## Conclusion

Reproduction is essential for the continuation of our species, as it ensures that parental genetic and epigenetic information are passed on to the next generation. Besides producing gametes, the reproductive system also provides the environment for the appropriate development of the embryo. New genomic and computational tools offer unique opportunities to study the intricate spatiotemporal regulatory mechanisms that are required for mammalian reproduction.

Like scRNA-seq, ST technologies hold tremendous potential for clinical applications. First, the identification of signaling pathways regulating human germ cell differentiation and proliferation will enable the development of protocols for human *in vitro* spermatogenesis—a technology that would have tremendous impact on fertility preservation. Second, spatially altered genes, cell types, and spatial neighborhoods under pathological conditions identified by ST technologies may be considered as markers for infertility, cancer, or other reproductive disorders. Finally, CCC through LR interactions revealed by ST technologies may also serve as new therapeutic targets for either treating reproductive disorders or developing novel contraceptive approaches.

In summary, our review discusses the novel biological insights that have been revealed by studies using ST technologies, while also shedding light on what is yet to come. We hope that this review will provide reproductive biologists and clinicians with a much-needed update on the state of art of ST technologies. This review may also facilitate the adoption of cutting-edge spatial omics technologies in both basic and clinical reproductive research.

## Data Availability

No new data were generated or analyzed in support of this research.

## Abbreviations list

AMMy: Anti-mesometrial myometrium
Asb4: Ankyrin repeat and SOCS box-containing 4
C7: Complement C7
camk2g1: Calcium/calmodulin-dependent protein kinase II gamma 1
CCC: Cell–cell communication
CDHR3: Cadherin-related family member 3
CFD: Complement factor D
CRISPR: Clustered regularly interspaced short palindromic repeats
CSCC: Cervical squamous cell carcinoma
Cxcl14: Chemokine (C-X-C motif) ligand 14
dazl: Deleted in azoospermia-like
Dio2: Deiodinase, iodothyronine, type II
ECM1: Extracellular matrix protein 1
ER: Excellent responders
ETV5: ETS variant transcription factor 5
EVTs: Extravillous trophoblast cells
Ezh2: Enhancer of zeste homolog 2
FAM183A: Family with sequence similarity 183 member A
FAP: Fibroblast activation protein alpha
FMI: Fetal–maternal interface
FOXJ1: Forkhead box J1
Fut9: Fucosyltransferase 9
GT: Glandular trophoblast cells
H2-Ab1: Histocompatibility 2, class II antigen A, beta 1
Habp4: Hyaluronan binding protein 4
HGSC: High-grade serous ovarian carcinoma
Hopx: HOP homeobox
Igfbp5: Insulin-like growth factor-binding protein 5
Il1b: Interleukin 1 beta
ISH: *In situ* hybridization
ISS: *In situ* sequencing
KO: Knockout
KRT10: Keratin 10
L1TD1: LINE1-type transposase domain containing 1
LCM: Laser capture microdissection
Lcn2: Lipocalin 2
LGR5: Leucine-rich repeat-containing G protein-coupled receptor 5
Malat1: Metastasis-associated lung adenocarcinoma transcript 1
MD: Mesometrial decidua
MERFISH: Multiplexed error-robust fluorescence *in situ* hybridization
MMP11: Matrix metallopeptidase 11
MMy: Mesometrial myometrium
Mt3: Metallothionein 3
myCAFs: Cancer-associated myofibroblasts
NGS: Next-generation sequencing
NOTCH2: Notch receptor 2
NR2F2: Nuclear receptor subfamily 2 group F member 2
Pdyn: Prodynorphin
PDZ: Primary decidual zone
PIFO: Primary cilia formation
PIWIL4: Piwi-like RNA-mediated gene silencing 4
PR: Poor responder
RCA: Rolling circle amplification
SCGB2A2: Secretoglobin family 2A member 2
scRNA-seq: Single-cell RNA sequencing
SDZ: Secondary decidual zone
seqFISH: Sequential single-molecule fluorescence *in situ* hybridization
Slc5a7: Solute carrier family 5, member 7
Smcp: Sperm mitochondria-associated cysteine-rich protein
smFISH: Single-molecule fluorescence *in situ* hybridization
SOX9: SRY-box transcription factor 9
ST: Spatial transcriptomics
STARmap: Spatially resolved transcript amplicon readout mapping
Stereo-seq: Spatial enhanced resolution omics-sequencing
Sult1d1: Sulfotransferase family 1D, member 1
TDZ: Transition decidual zone
TG: Trophoblast giant cells
TPPP3: Tubulin polymerization promoting protein family member 3
udStr: Undifferentiated stroma
Vsnl1: Visinin-like 1
VSZ: Vascular sinus zone
WNT7A: Wnt family member 7A
WT: Wild type
